# Automated Quantitative Analysis of Blood Flow in Extracranial–Intracranial Arterial Bypass Based on Indocyanine Green Angiography

**DOI:** 10.3389/fsurg.2021.649719

**Published:** 2021-06-11

**Authors:** Zhuoyun Jiang, Yu Lei, Liqiong Zhang, Wei Ni, Chao Gao, Xinjie Gao, Heng Yang, Jiabin Su, Weiping Xiao, Jinhua Yu, Yuxiang Gu

**Affiliations:** ^1^School of Information Science and Technology, Fudan University, Shanghai, China; ^2^Department of Neurosurgery, Huashan Hospital, Fudan University, Shanghai, China

**Keywords:** automatic blood flow quantitative analysis, vessel segmentation, image registration, HS optical flow, indocyanine green angiography

## Abstract

Microvascular imaging based on indocyanine green is an important tool for surgeons who carry out extracranial–intracranial arterial bypass surgery. In terms of blood perfusion, indocyanine green images contain abundant information, which cannot be effectively interpreted by humans or currently available commercial software. In this paper, an automatic processing framework for perfusion assessments based on indocyanine green videos is proposed and consists of three stages, namely, vessel segmentation based on the UNet deep neural network, preoperative and postoperative image registrations based on scale-invariant transform features, and blood flow evaluation based on the Horn–Schunck optical flow method. This automatic processing flow can reveal the blood flow direction and intensity curve of any vessel, as well as the blood perfusion changes before and after an operation. Commercial software embedded in a microscope is used as a reference to evaluate the effectiveness of the algorithm in this study. A total of 120 patients from multiple centers were sampled for the study. For blood vessel segmentation, a Dice coefficient of 0.80 and a Jaccard coefficient of 0.73 were obtained. For image registration, the success rate was 81%. In preoperative and postoperative video processing, the coincidence rates between the automatic processing method and commercial software were 89 and 87%, respectively. The proposed framework not only achieves blood perfusion analysis similar to that of commercial software but also automatically detects and matches blood vessels before and after an operation, thus quantifying the flow direction and enabling surgeons to intuitively evaluate the perfusion changes caused by bypass surgery.

## Introduction

The superficial temporal artery to middle cerebral artery (STA-MCA), first reported by Spetzler and Martin in 1986 (1), has been widely used in blood flow reconstruction for cerebral hemorrhages or ischemia and complex intracranial aneurysms (2–5). STA-MCA alters the cerebral blood flow (CBF) perfusion of patients, so methods are needed to analyze the changes in blood flow before and after an operation to assist surgeons in adjusting follow-up therapeutic schedules at any time according to the operation effect. Raabe et al. (6, 7) first demonstrated that the dynamic flow of indocyanine green (ICG) in anastomotic vessels can help in determining the patency and direction of intraoperative blood flow (8–10). A host of studies (11–14) have proven that intraoperative ICG video angiography (ICG-VA) and current commercial software can be employed to generate time-delayed color maps to monitor regional CBF and evaluate improvements in cortex perfusion around the anastomotic site after bypass. As an image postprocessing software, this software is unable to achieve continuous real-time and dynamic analysis of CBF and cannot quantify the flow direction of each blood vessel. Therefore, it is clinically helpful to develop a fully automated quantitative blood flow analysis system that can be used to measure the real-time flow direction based on the original ICG-VA videos and to visually compare the difference in flow perfusion by correcting the images before and after the operation.

The proposed automatic and quantitative blood flow analysis algorithm consists of three parts, namely, vessel segmentation, preoperative and postoperative vessel registrations, and blood flow analysis. Quantifying blood flow is the main task, and vessel segmentation and registration are prerequisite tasks. There are many blood vessels with large diameter differences and low definition, and we use the UNet deep neural network (15) to carry out classification at the pixel level. The main factors that cause image differences before and after surgery are as follows (16): (i) changes in the position of the microscope lens and movements along the X-, Y- and Z-axes; (ii) variations in camera interior parameters, such as focal length and resolution; and (iii) perfusion changes caused by bypass surgery. To visually compare the differences in blood perfusion before and after bypass surgery, it is essential and necessary to register the target (preoperative) image to the direction of the reference (postoperative) image. Vessel features are widely used in common feature-based registration methods (17–20). Since the rotation-invariant distance of the scale-invariant feature transform (SIFT) remains invariant to rotation, scaling, and brightness, this kind of method is an optimal choice for this work. The optical flow method can accurately identify the position of a moving object without knowing the information of the scene. Optical flow is the instantaneous velocity of the pixel movement of a spatially moving object on an observation imaging plane. It uses the change in a pixel in the time domain in an image sequence and the correlation between adjacent frames to calculate the motion information of objects between adjacent frames (21). Experiments have revealed that for any two adjacent frames in ICG-VA, the changes in the brightness and motion distance of pixels corresponding to blood were slight and satisfy the conditions necessary to apply the optical flow method (22). An improved Horn–Schunck (HS) optical flow method (22) is used in this study to determine the flow direction in ICG-VA to evaluate the practical application of our process with ICG-VA in CBF reconstruction.

**Figure 1:**
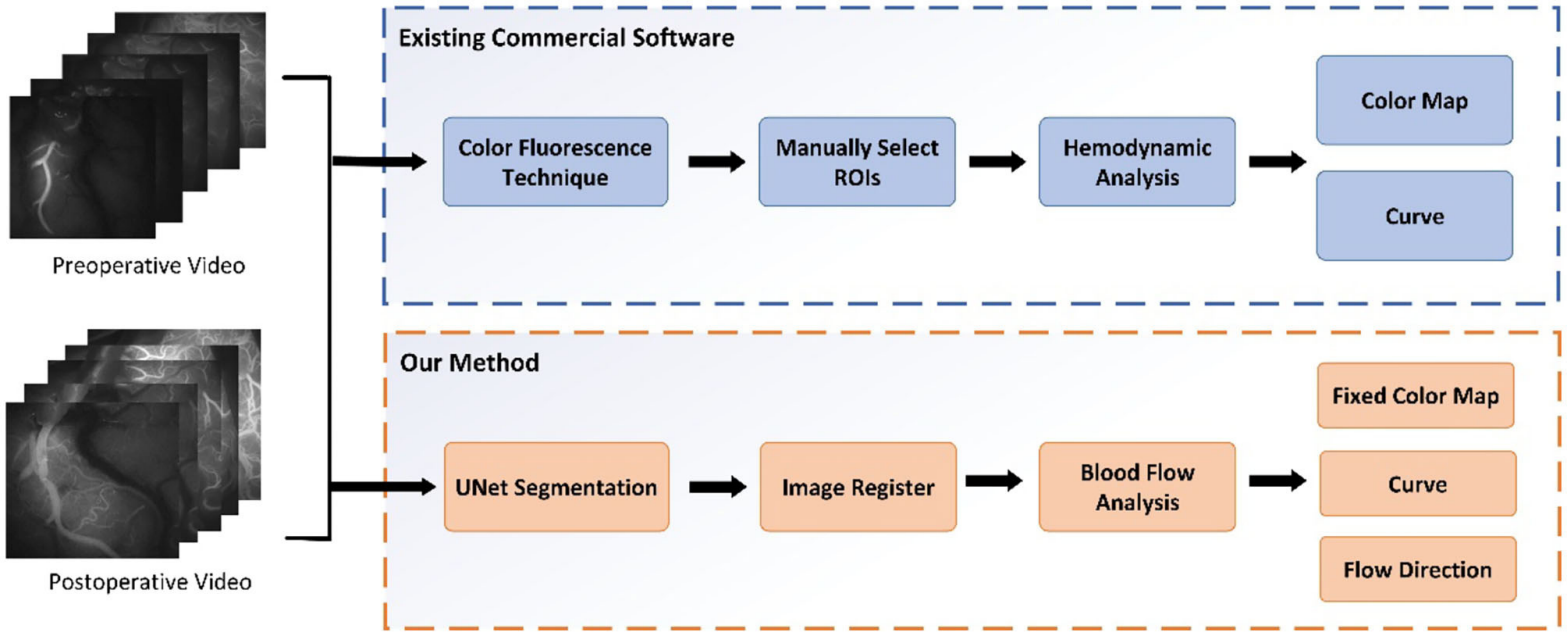
The process of this study and the process of existing commercial software.

The main contributions of our paper are as follows. (i) An automatic quantitative blood flow analysis process, which can perform vessel segmentation, vessel registration, and flow direction analysis, is proposed. (ii) Based on the registration of the video images before and after an operation, an evaluation of the blood perfusion changes can be obtained. (iii) An algorithm for tracking the blood flow direction in videos obtained with ICG-VA based on the optical flow field is proposed. Based on the change in video brightness, a blood perfusion color map and time–luminance curve are obtained. Figure 1 shows the comparison process between this study and existing commercial software.

## Materials and Methods

### Patients and Surgical Procedures

One hundred twenty patients who were diagnosed with moyamoya disease (MMD) and underwent STA-MCA bypass surgery at two medical centers (the North Campus of Huashan Hospital and Huashan Clinical Medical Center) from October 2018 to June 2020 were retrospectively investigated. All of the patients met the Chinese guidelines for the diagnosis and treatment of MMD set by the Stroke Prevention Project Committee, National Health and Family Planning Commission, China. STA-MCA bypass surgery was performed with the OPMI PENTERO 900 and OPMI PENTERO 800 microscopes with embedded blood flow analysis commercial software. This study was approved by the Institutional Review Board of Huashan Hospital, Fudan University. All participants provided informed consent.

**Figure 2:**
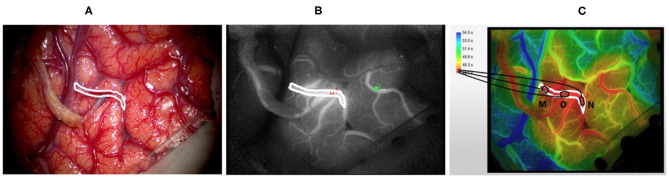
Displays the intraoperative findings of patients with moyamoya disease who received superficial temporal artery to middle cerebral artery (STA-MCA) bypass surgery and the indocyanine green video angiography (ICG-VA) images generated by commercial software. **(A)** The microscope field showing the completion of the STA-MCA anastomosis. **(B)** The image of ICG-VA. **(C)** The postprocessed blood flow analysis image of commercial software. The white frame represents the recipient vessel; M, N, and O are three areas of the recipient vessel (O denotes the bypass grafting site).

**Figure 3:**
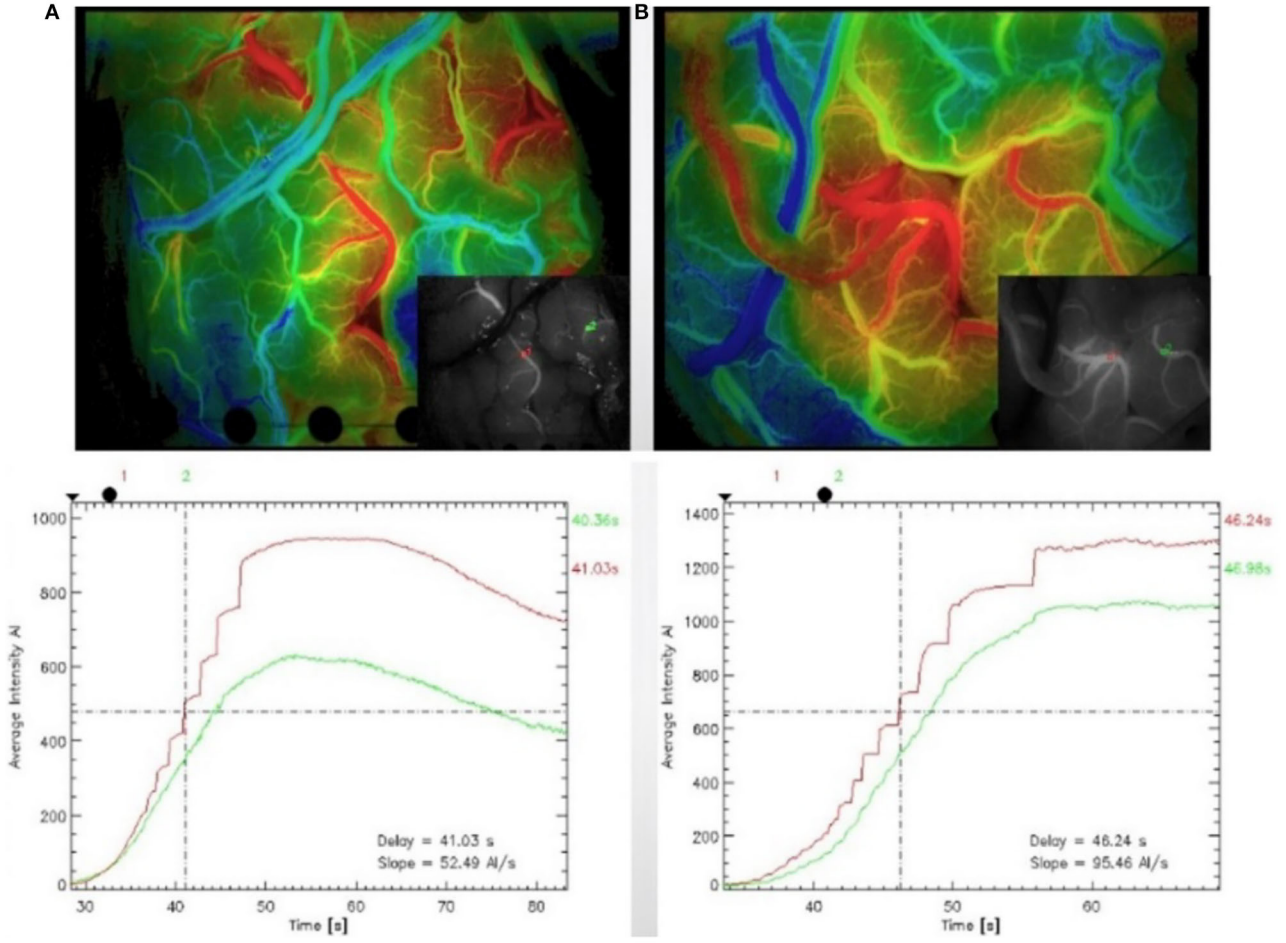
Indocyanine green (ICG) was performed after craniotomy to identify the recipient artery in the operative area **(A)**. The region of interest (ROI) was set at the artery branch, and blood flow analysis was carried out again after the anastomosis to confirm the patency of the vessel after the anastomosis and compare the blood flow changes in the previously selected regions **(B)**.

All of the surgeries were performed by experienced neurosurgeons. ICG was performed after the craniotomies to determine the candidate recipient artery based on the diameter and length of the STA graft. During the imaging process, a microscope integrated with a near-infrared light-emitting device and a fluorescence acquisition system was aligned with the operating field (Figure 2A), and the ICG was diluted with 20 ml of isotonic saline and injected into patients through a peripheral intravenous bolus. ICG fluorescence signals with a length of approximately 1 min were collected with a microscope and then processed by a computer to generate black and white angiography videos (Figure 2B). Subsequently, commercial blood flow analysis software built into the microscope operating platform was used to conduct postprocessing analysis, and the results are presented in color images (Figure 2C). The sections where the contrast agent first and last passed are red and blue-purple, respectively. The regions of interest (ROIs) at the artery branch were set to calculate the delay time and plot the intensity curve (Figure 3). The same procedure was performed again instantly after the surgery to confirm the bypass patency and compare it with the preoperative blood flow changes in the selected regions (Figure 3B). Taking a recipient vessel after bypass as an example, it is not easy to distinguish similar colors with the naked eye because the entire vessel is red (the white framed sections in Figures 2A–C). In this study, three ROIs, namely, M, O and N, were set up for a vessel, and the RGB values of these three parts were obtained by a computer (R denotes red, G denotes green, and B denotes blue). The “gold standard” for blood flow direction was determined by finding the corresponding position on the color bar. Figure 2C shows blood flows from bypass graft sites O to M and N.

### Automated Quantitative Analysis of Blood Flow

**Figure 4:**
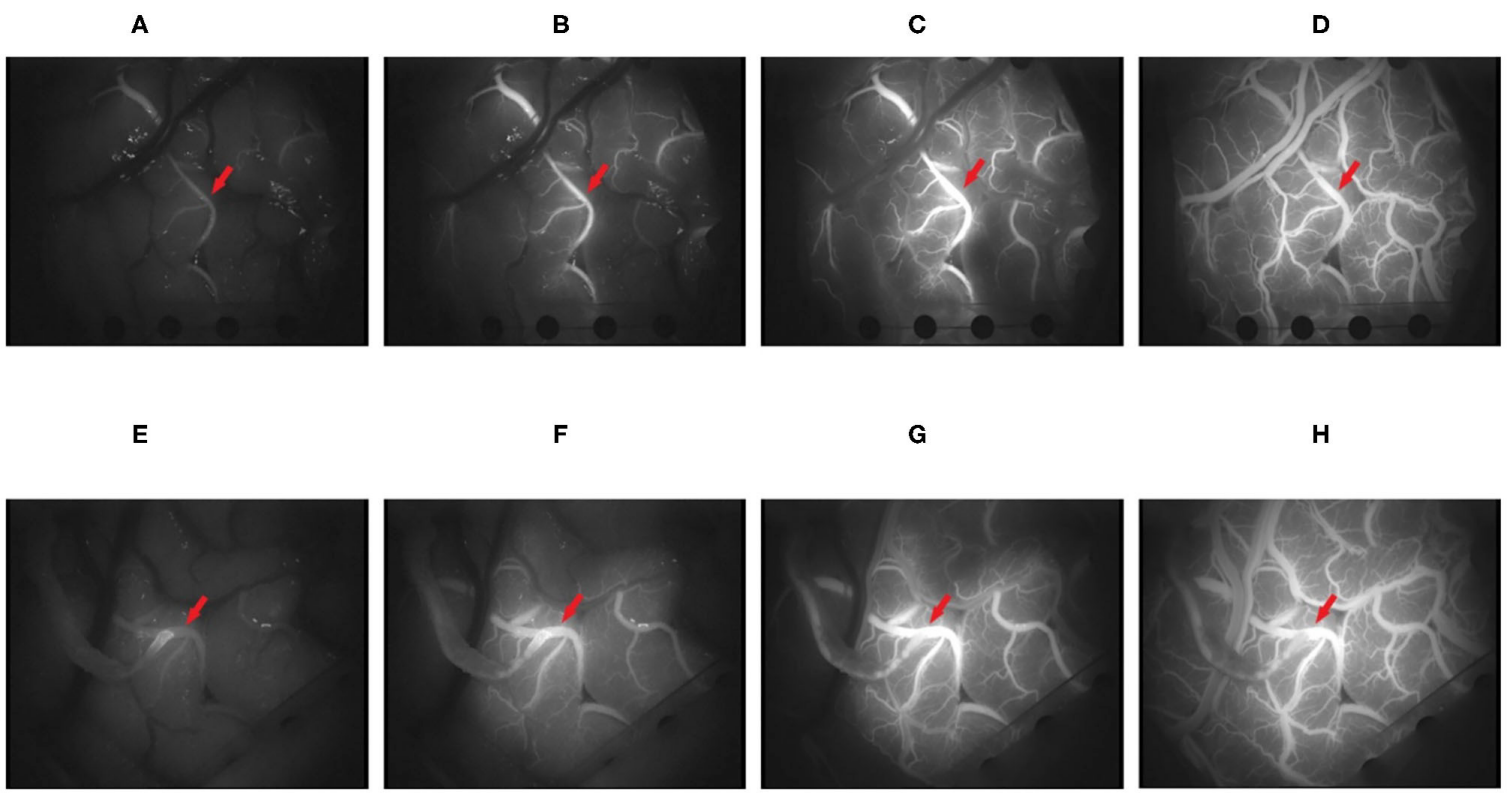
It shows the changes in the brightness of indocyanine green (ICG) in four frames of video intercepts before and after superficial temporal artery to middle cerebral artery (STA-MCA) anastomosis in patients with moyamoya disease. **(A–D)** The 100, 150, 250, and 400th frames before operation. **(E–H)** The 100, 150, 250, and 400th frames after the operation (the red arrow points to the recipient vessel).

This study developed a universal and repeatable algorithm to automatically provide quantitative analyses of blood flow and compared the results with those of commercial software. The video (MPG format) of the change process of the intracranial receptor vessels in ICG-VA from dark to light was captured with a frame rate of 25 frames/s and an image resolution of 720 ×576. Figure 4 shows four randomly selected frames from the postoperative video of a patient. The fluorescent agent in the initial position of the blood flow is illuminated first and reaches the maximum brightness first. The change in brightness from dark to bright reflects the order in which the blood flow passes through the receptor vessel.

#### Algorithm Framework

**Figure 5:**
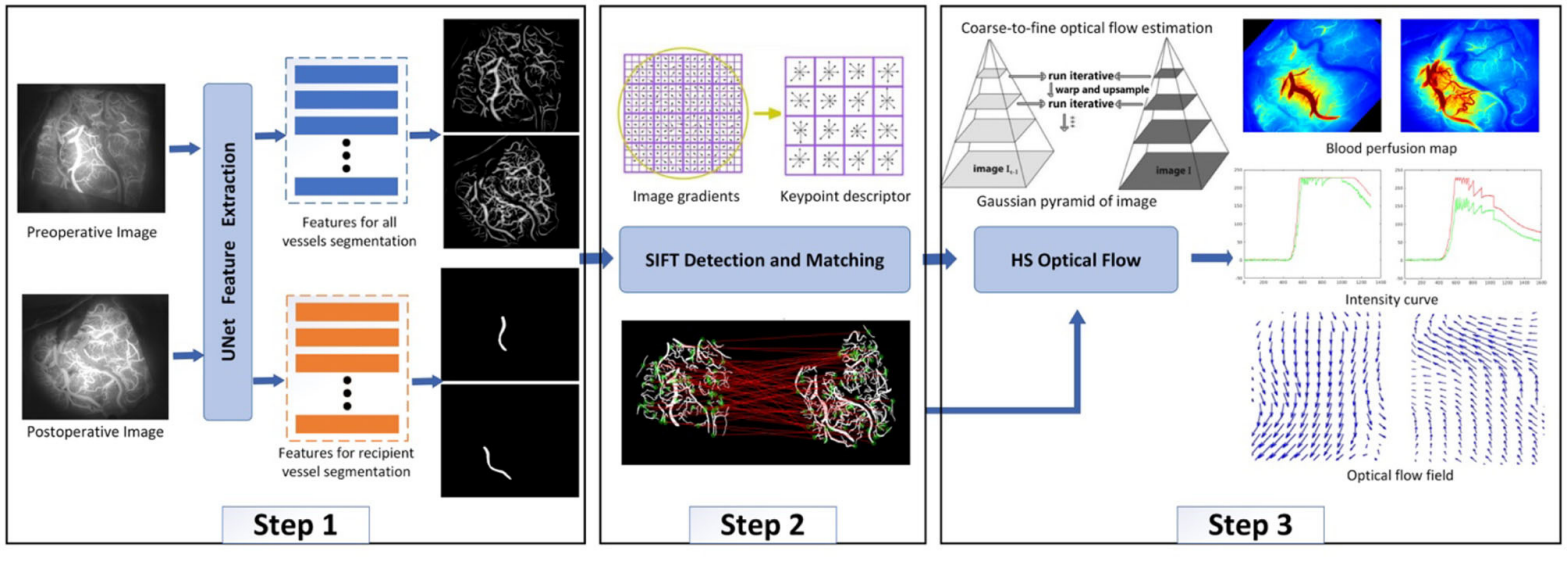
Algorithm framework.

After ICG-VA is decomposed into multiple frames, it can be found that the cerebral vessels are piecewise-linear, and their width and branches are visible in the image. Therefore, a multitask UNet model was designed in this study to simultaneously segment all vessels and recipient vessels. The purpose of separating the recipient vessel was to observe the patency of blood flow near the anastomosis site. The next step was to match images from before and after an operation to avoid differences in blood flow results caused by image differences. By detecting the SIFT descriptor of the vessels, the preoperative images were homographically changed and aligned with the postoperative images. The third step was to measure the blood flow based on the HS optical flow field. Specifically, we measured the real-time direction of the blood flow in segmented vessels, drew a blood perfusion map and an intensity curve, and analyzed whether the blood flow direction of recipient vessels changed after the operation. The algorithm framework is shown in Figure 5.

#### Vessel Segmentation

**Figure 6:**
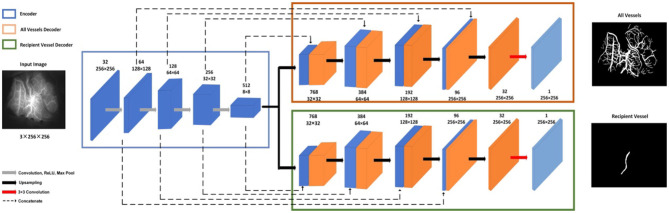
Vessel segmentation network.

The multitask UNet model is a network of encoder–decoder structures. On the left is the contracting encoder path, which uses a typical CNN architecture, and on the right, there are two deconvolutional decoder paths (23). The encoder path includes four repeated layers with two successive 3 ×3 convolutions, nonlinear activation, and max pooling operations that halve the size of the feature map after each convolutional layer. The two decoder modules have the same structure, where at each level, the feature map is upsampled with 2 ×2 upconvolution. Then, the feature map from the corresponding layer in the encoder path is cropped and concatenated onto the upsampled feature map to retain multiscale features. This is followed by two successive 3 ×3 convolutions and nonlinear activation. In the final stage, an additional 1 ×1 convolution operation is applied to reduce the feature map to the required number of channels and produce the segmented image. The two decoder modules have different training tasks: one is to segment all vessels and the other is to segment recipient vessels only. The network structure is shown in Figure 6.

The total loss consists of two parts, namely, the negative log likelihood loss of all vessel segmentation tasks and the recipient vessel segmentation task; see formula (1).

Formula

(1) $$\begin{array}{r} L_{total}\;=\;\alpha L_{all}\;+\;\beta L_{receip} \end{array}$$

where α and β are scaling factors, and *L*_*all*_ and *L*_*receip*_ are defined by $L_{all}=\underset{x\epsilon \Omega }{\sum }logp_{all}\left(x;l_{all}\left(x\right)\right)$ and $L_{receip}=\underset{x\epsilon \Omega }{\sum }logp_{receip}\left(x;l_{receip}\left(x\right)\right)$, respectively. *L*_*all*_ and *L*_*receip*_ represent the classification error function of all segmented vessels and the segmented recipient vessels, respectively. *x* denotes the pixel position in the image space Ω. *p*_*all*_(*x*; *l*_*all*_) represents the predicted probability of the true label *l*_*all*_ after applying the softmax activation function. *p*_*receip*_(*x*; *l*_*receip*_) represents the predicted probability of the real label *l*_*receip*_ after applying the softmax activation function.

#### Image Registration

The basic idea of the matching algorithm is to map and geometrically align the segmented vessel images before and after the surgery. SIFT, which is invariant to rotation, scaling, and noise (24, 25), is employed to detect the features of the vessel image. Difference of Gaussian (DOG) (26) used Gaussian difference convolution to detect the local maximum or minimum value of the image to obtain stable SIFT keypoints. Then, a gradient histogram will be created for each keypoint to achieve rotation invariance of the image. The histogram is composed of the gradient orientation of the sampling points in the neighborhood around the keypoint. The final step of SIFT is to obtain the keypoint descriptor based on the previous steps, which is a 128-dimensional feature vector involving location, scale, and orientation.

After the SIFT descriptor is obtained, the nearest neighbor search was used to match the image pairs (27). Specifically, for the invariant descriptor vector, the nearest neighbor is defined as the keypoint with the minimum Euclidean distance. An effective measure for matching verification is to calculate the distance ratio between the nearest neighbor and the next nearest neighbor. We set this ratio as 0.8 based on many experiments.

#### Horn–Schunck Optical Flow Measurement

After the vessels were automatically acquired in the first two steps, the HS optical flow method was used to track the motion direction of each pixel of the recipient vessel between adjacent frames. First, a Gaussian filter was used to construct the original ICG image pyramid, which was decomposed into four layers in this study. Second, the HS optical flow method was employed to calculate the optical flow of the layered images. The motion direction of each pixel along the X- and Y-axes can be calculated, that is, from left to right, from top to bottom, or vice versa. The final direction of the blood flow of the recipient vessel can be obtained by analyzing the flow directions of all pixels of the vessel along the X- and Y-axes in ICG-VA.

The HS optical flow method uses formula (2) to describe that the brightness of the recipient vessel pixels in the *t*-th frame is consistent with that in the (*t* + Δ*t*)-th frame.

Formula

(2) $$\begin{array}{r} I\left(x,y,t\right)\;=\;I\left(x\;+\;\Delta x,y\;+\;\Delta y,t\;+\;\Delta t\right) \end{array}$$

Δ*x* represents the movement distance of the pixel along the X-axis, Δ*y* denotes the motion distance of the pixel along the Y-axis, Δ*t* indicates the time difference between adjacent frames, and *I*(*x, y, t*) is the brightness value of pixel (*x, y*) at time *t*. The same pixel has similar brightness levels in adjacent frames and slight changes in motion distance. The motion distance of the pixel is constrained by introducing the smooth energy function *E*_*s*_, which is given in formula (3).

Formula

(3) $$\begin{array}{r} E_{s}=\iint \left[{\left(u_{x}\right)}^{2}+{\left(u_{y}\right)}^{2}+{\left(v_{x}\right)}^{2}+{\left(v_{y}\right)}^{2}\right]dxdy \end{array}$$

where *u*_*x*_ represents the partial derivative of *u* along the X-axis, *u*_*y*_ denotes the partial derivative of *u* along the Y-axis, *v*_*x*_ denotes the partial derivative of *v* along the X-axis, and *v*_*y*_ denotes the partial derivative of *v* along the Y-axis. The energy formula (4) for calculating the optical flow field is obtained by combining formula (2) and formula (3) and employing the iterative calculation method.

Formula

(4) $$\begin{array}{l} \min\;E\;=\iint \{{\left(I_{x}u\;+\;I_{y}v\;+\;I_{t}\right)}^{2}\;+\;\lambda [{\left(u_{x}\right)}^{2}\;+\;{\left(u_{y}\right)}^{2}\\ \;+\;{\left(v_{x}\right)}^{2}+{\left(v_{y}\right)}^{2}]\}dxdy \end{array}$$

λ denotes the smooth coefficient; the greater λ is, the higher the smoothness and the higher the accuracy of the estimation (22). After many experiments, it is found that when the value of λ is 2, we can obtain the best results.

The iterative method is a functional extreme value problem, which can be solved by the Euler–Lagrange equation to obtain the iterative formula (5),

Formula

(5) $$\begin{array}{r} u^{k+1}={\bar{u}}^{k}-\frac{I_{x}\left(I_{x}{\bar{u}}^{k}+I_{y}{\bar{v}}^{k}+I_{t}\right)}{\lambda^{2}+I_{x}^{2}+I_{y}^{2}} \end{array}$$

(6) $$\begin{array}{r} v^{k+1}={\bar{v}}^{k}-\frac{I_{y}\left(I_{x}{\bar{u}}^{k}+I_{y}{\bar{v}}^{k}+I_{t}\right)}{\lambda^{2}+I_{x}^{2}+I_{y}^{2}} \end{array}$$

where $\bar{u}$ and $\bar{v}$ are the mean values of *u* and *v*, respectively, and the initial values are 0. The values of *u* and *v* are calculated iteratively in turn. When the condition is satisfied, i.e., when the difference between the energy function values of two consecutive times is less than the given threshold 0.1, the iteration is terminated and the optical flow values *u* and *v* are obtained.

## Results

In this section, we first introduce the experimental settings, evaluation indicators, and experimental results for vessel segmentation and show the experimental results of registration. Then, we elaborate the evaluation criteria of the optical flow method. Finally, we compare the algorithm in this study with commercial software. The algorithm was performed with Python and MATLAB. The experiments were conducted on an NVIDIA GeForce GTX 1080 GPU 6 GB RAM.

### Vessel Segmentation

#### Implementation Details

All models were implemented through PyTorch. We employed the Adam optimizer and set the batch size to 32. We used the plateau strategy to reduce the learning rate. At the 0, 20, and 100th iterations, the learning rate was set to 0.01, 0.001, and 0.0001, respectively, and the total number of iterations was set to 150.

Due to the limited number of training images, the dataset was augmented to avoid overfitting (28). First, the data were augmented through horizontal, vertical, and diagonal flips so that each image in the original dataset was augmented into four images. To improve the robustness of image segmentation, the same data augmentation was performed on the testing images (29, 30), which meant that each image was predicted five times. Then, we took the average value of the five predictions to obtain the final prediction map.

#### Segmentation Results

**Figure 7:**
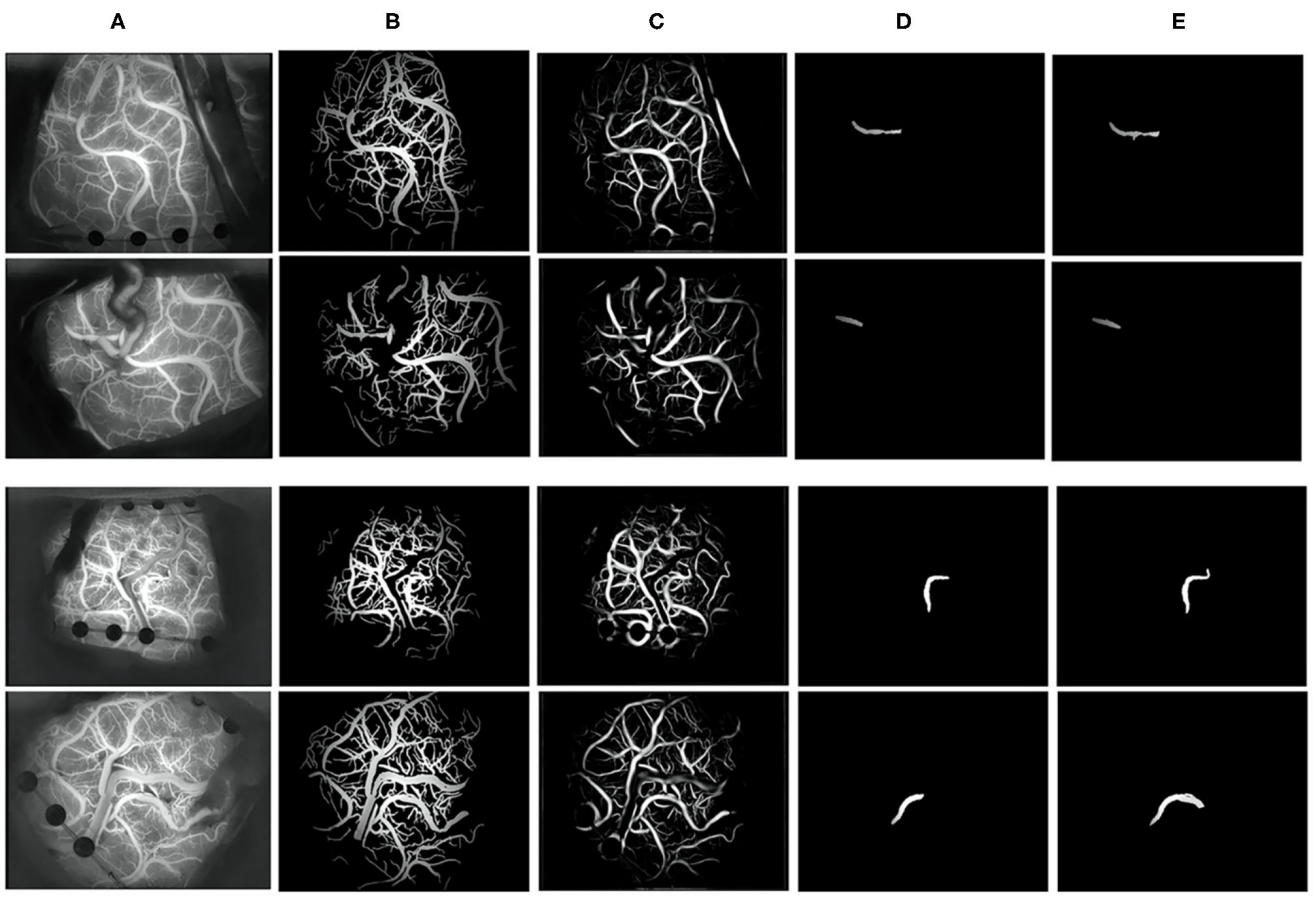
Vessel segmentation results. **(A)** The original image, **(B)** groundtruth, **(C)** the segmentation result of our algorithm, **(D)** the recipient vessel groundtruth, and **(E)** the segmentation result of the recipient vessel in this article.

We selected frames from ICG-VA in which the brightness is stable and no longer changes for segmentation. There were 240 images in total; 150 images were used for training, and 90 were used for testing. Two indicators, the Jaccard index and Dice coefficient (23), were used to evaluate segmentation performance. These two coefficients are used to compare the similarity and difference between two samples. The larger the Jaccard and Dice coefficients are, the higher the sample similarity is. We obtained a segmentation result with a Dice coefficient of 0.80 and a Jaccard coefficient of 0.73. Figure 7 shows some segmentation results.

### Image Registration

We objectively evaluated the overall performance of the registration algorithm. For image pairs that overlap in at least 35% of the vessel pixels and have at least 10 SIFT feature correspondences, the matching is considered successful. The success rate (31) is the ratio of the number of successfully matched image pairs to the number of all image pairs. Among 120 image pairs, 97 pairs were successfully matched in this paper, with the success rate was 0.81.

**Figure 8:**
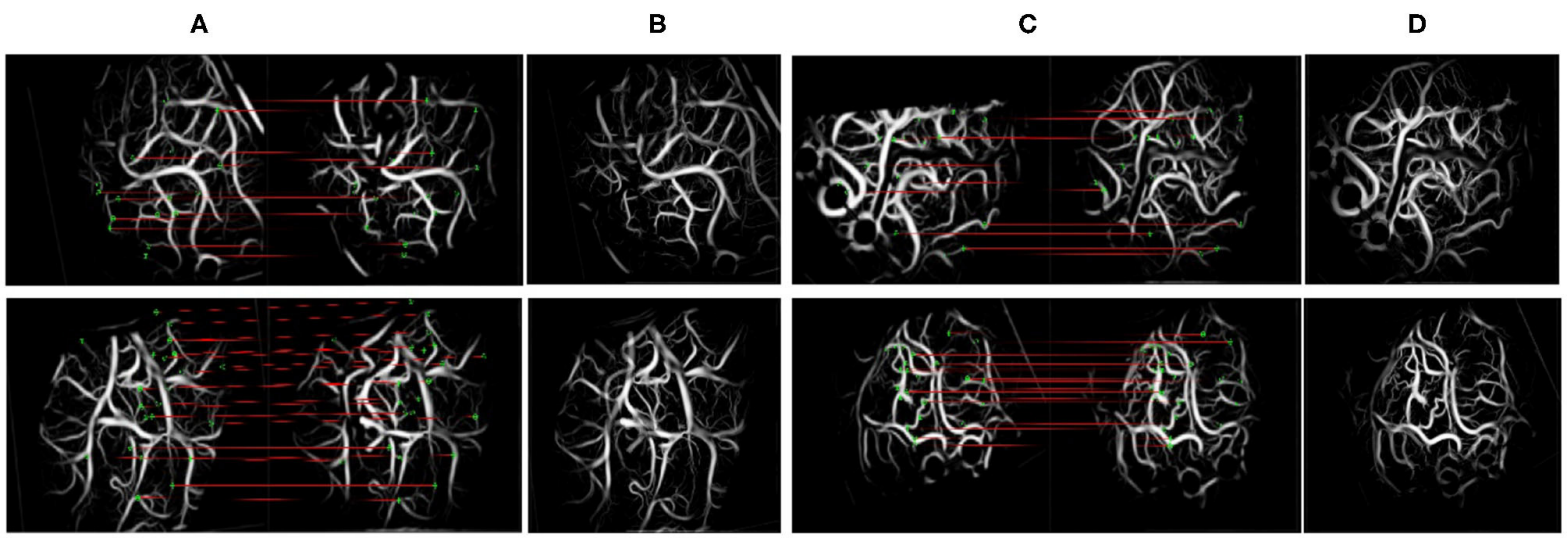
Scale-invariant feature transform (SIFT) feature and matching. The left side of **(A,C)** denotes the moving images, and the right side denotes the fixed images. **(A,C)** The results of keypoint matching. **(B,D)** The fusion images registered by our method.

Figure 8 shows the matching results after extracting the SIFT features from the blood vessel segmentation map. In terms of the upper left corner image pair, 215 and 276 keypoints were detected, and 27 matching pairs were generated through the algorithm. The green point represents the SIFT features on the matching pairs. The third and sixth columns of Figure 8 show an overlapping image that contains a preoperative image and a postoperative image after the homograph changes so that the matching results can be evaluated more intuitively. This matching algorithm can address translation, rotation, and scaling.

### Quantitative Analysis of Blood Flow

#### Interpretation of Results

The optical flow method and commercial software were used to interpret the results of 240 preoperative and postoperative videos of 120 patients. If the optical flow method and commercial software are consistent in the interpretation of the flow direction (forward and reverse) in the preoperative videos and the flow direction (forward, reverse, and from the middle to both sides) in the postoperative videos, the result is deemed to be correct. The optical flow method and commercial software were used to interpret whether the direction of the blood flow of each patient was changed after the operation. If the two methods showed that the direction of blood flow was changed, the result would be considered consistent.

SPSS 18.0 software was adopted for statistical analyses of the data. The measurement data that conform to a normal distribution are represented by χ ± s, and the enumeration data are expressed as frequencies and percentages (%). To verify the validity of this research method, the analysis results of the HS optical flow method were compared with those generated by commercial software. A kappa of <0.2 was considered a slight agreement, a kappa of 0.2 < κ <0.4 was considered a fair agreement; a kappa of 0.4 < κ <0.6 was considered a moderate agreement; a kappa of 0.6 < κ <0.8 was considered a substantial agreement; and a kappa of κ > 0.8 was considered an almost perfect agreement. A *P*-value of <0.05 was considered statistically significant.

#### Flow Direction Measurement

**Figure 9:**
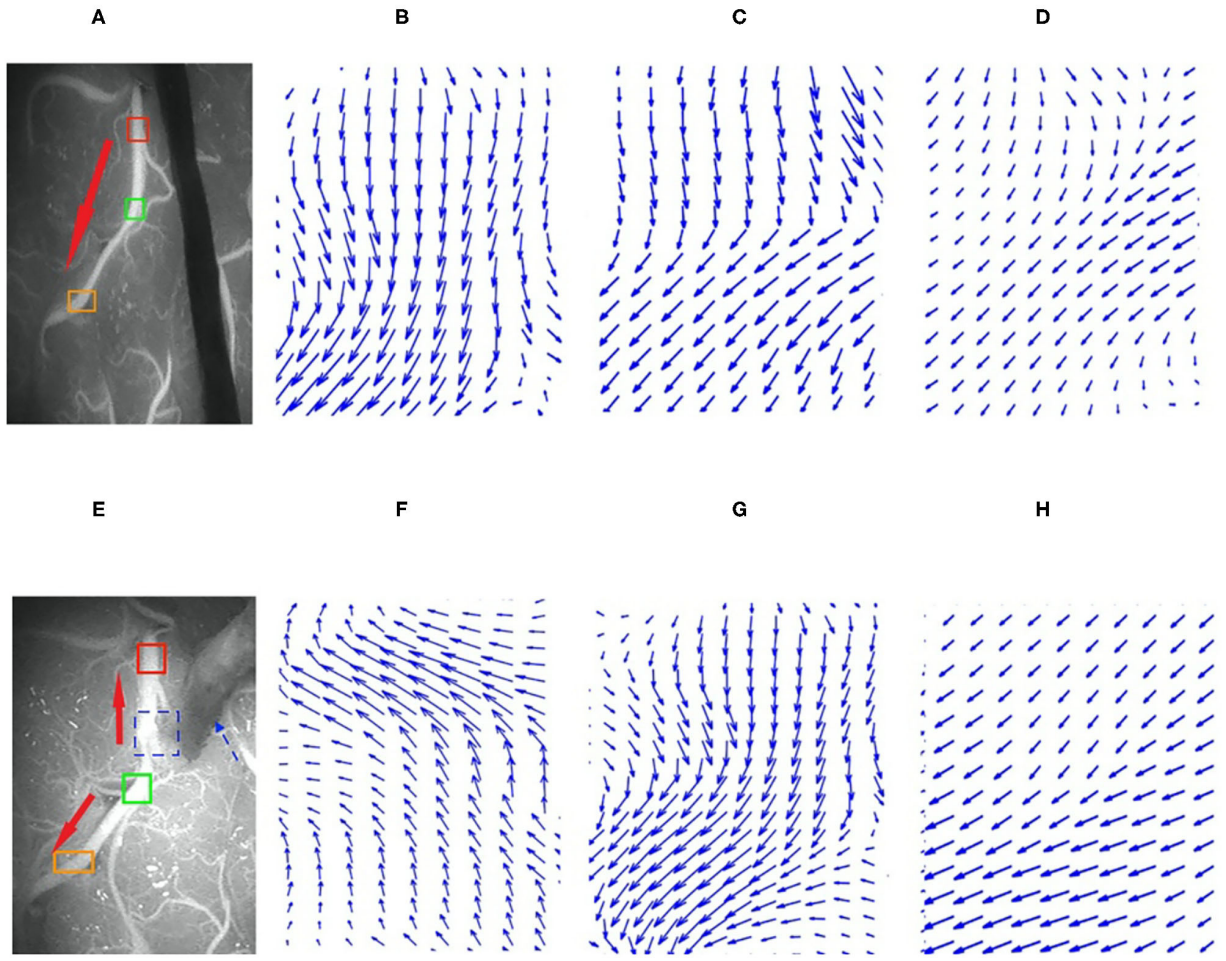
Preoperative and postoperative indocyanine green (ICG) images and their local optical flow vector diagrams. **(A–D)** Preoperative; **(A)** the ICG image; **(B–D)** the optical flow vectors in the red, green, and orange frames, respectively. **(E–H)** Postoperative; **(E)** is ICG image; **(F–H)** the optical flow vectors of the red, green, and orange frames, respectively. Red, green, and orange frames denote the three arbitrarily selected sections in the recipient vessel.

The HS optical flow method and commercial software were highly consistent in their interpretations of the flow direction in the videos before and after surgery. The kappa values obtained before and after the operation were 0.775 and 0.768, respectively, with *P* <0.01 (see Table 1). The analysis results from 107 preoperative videos and 104 postoperative videos of 120 patients obtained through the HS optical flow method were consistent with those of the commercial software. Figure 9 shows the optical flow vector diagram of four frames obtained by ICG-VA of a case, and the vector direction reflects the movement direction of the pixel points. The dotted blue arrow points to the recipient vessel, and the dotted blue frame denotes the anastomotic stoma. In this case, the commercial software knows that the correct flow direction before operation is from the top red box to the middle green box and finally to the bottom orange box. The correct flow direction after the operation is from the middle green box to the top red box and the bottom orange box at both ends. The red arrows in Figures 9A,E indicate the correct flow direction.

**Table 1:** Consistency of HS optical flow method and commercial software in the interpretation of blood flow direction.

**HS optical flow**	**Commercial software**			**Total**
	**Positive flow**	**Reverse flow**	**Middle to both sides**	
**Consistency in preoperative video**				
Positive flow	74	4	–	78
Reverse flow	8	34	–	42
Middle to both sides	–	–	–	–
Total	82	38	–	120
**Consistency in postoperative video**				
Positive flow	64	3	2	69
Reverse flow	1	18	3	22
Middle to both sides	5	2	22	29
Total	70	23	27	120
**Recipient blood vessel direction after bypass surgery**				
	Changed	Unchanged		
Changed	48	10		58
Unchanged	7	55		62
Total	55	65		120
**Symmetric measures**				
	Value	Standardized error	Approximate T.	Sign.
Kappa in preoperative	0.775	0.061	8.517	0.000
Kappa in postoperative	0.768	0.053	11.493	0.000
Kappa in direction change	0.716	0.064	7.852	0.000

*HS, horn–schunck*.

The analysis results of the commercial software and the HS optical flow method revealed that after the operation, the numbers of patients with changes in the blood flow direction of the recipient vessel were 55 and 58, respectively, and the kappa value was 0.716 and *P* = 0.002 (see Table 1).

#### Color Map and Curve

**Figure 10:**
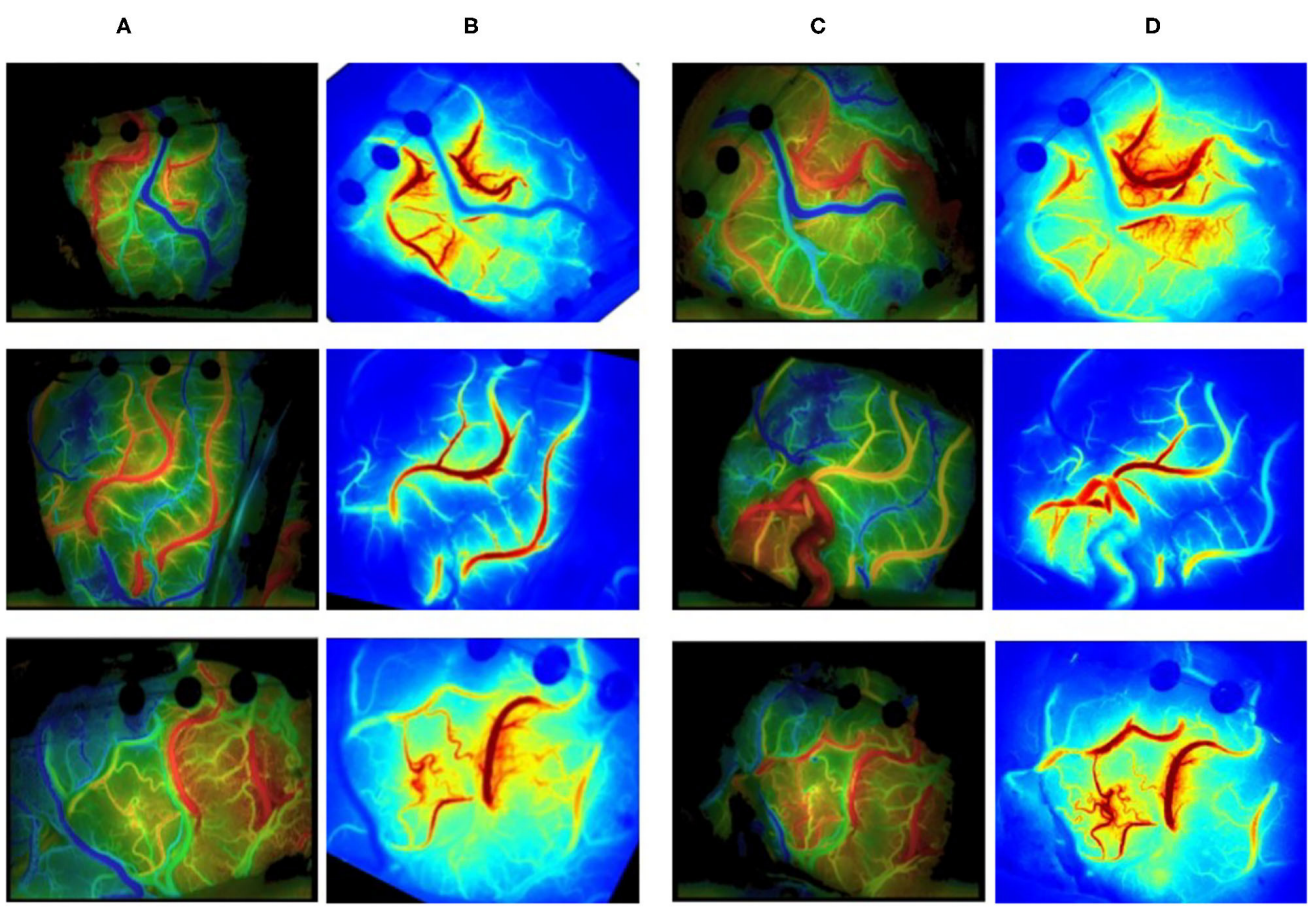
Blood flow perfusion color map. **(A, C)** The preoperative and postoperative blood perfusion color maps of three patients by commercial software. **(B, D)** The preoperative and postoperative perfusion maps by our method. **(B)** Registered based on **(D)**.

**Figure 11:**
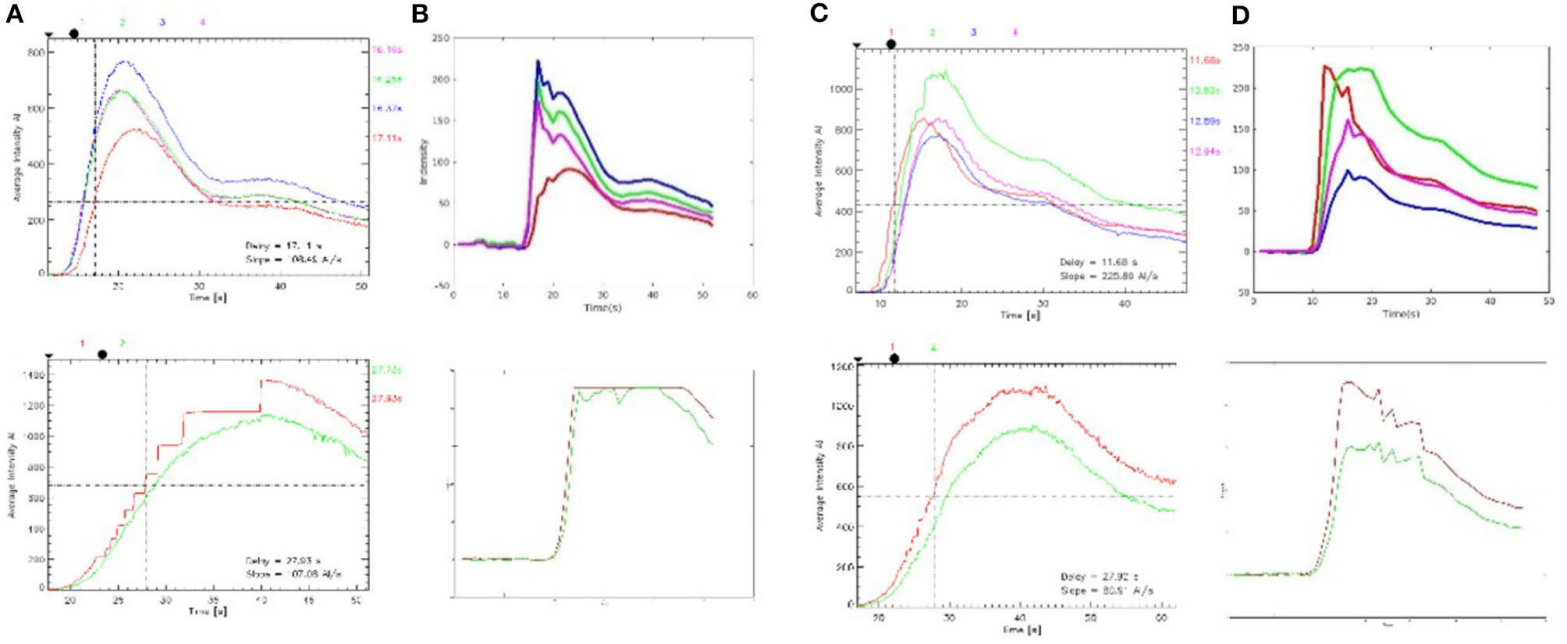
Time–brightness curve. **(A, C)** The region of interest (ROI) time–luminance curves calculated by commercial software. **(B, D)** The ROI time–luminance curves calculated by this method.

The luminance values of the ROIs selected in the commercial color map changed over time was calculated to obtain the time–luminance curve and assigned RGB color values to each pixel of the ICG-VA image based on the brightness value. Compared with the commercial software, it can be found that the curve obtained in this paper has a similar trend (see Figure 10), and the blood perfusion information reflected by the color map drawn in this paper is also similar to that of the software (see Figure 11).

The time required to reach the maximum brightness values of the ROIs selected by the commercial software is calculated by our method and compared with the delay time calculated by the commercial software. There were 127 ROIs in 120 preoperative videos and 178 ROIs in 120 postoperative videos. First, the normal Q–Q plot test was used to verify that the difference in the data obtained by the two methods obeyed a normal distribution, which met the precondition of the *T*-test. Then, a paired sample *T*-test was used to judge whether the time difference between the delay times calculated by these two methods was statistically significant. The results of the paired sample *T*-test before and after surgery are shown in Table 2; *P* = 0.745 before surgery and *P* = 0.671 after surgery. *P* > 0.05, which indicates that there was no difference between the two methods, and they are highly similar.

**Table 2:** Preoperative and postoperative paired samples test.

**Our method—software**	**Paired differences**					**t**	**df**	**Sig.(2-tailed)**
	**Mean**	**Std. deviation**	**Std. error mean**	**95% confidence interval**				
				**Lower**	**Upper**			
Preoperative	−0.02394	0.25809	0.02290	−0.06926	0.2139	−1.045	126	0.298
Postoperative	−0.01910	0.25363	0.01901	−0.05662	0.01842	−1.005	177	0.316

## Discussion

In this study, UNet, SIFT, nearest neighbor search, and the HS optical flow method were innovatively integrated into the automatic blood flow quantitative analysis system. We discussed three basic tasks: image segmentation, registration, and blood flow direction measurement. Blood flow measurements were used to compare the blood flow changes in the recipient vessel before and after the surgery. Due to the deformation of the captured ICG-VA images, it is necessary to register the images first. As the matching algorithm requires the successful segmentation of vessels in ICG-VA images, this study encountered many technical challenges. Experiments have confirmed that the results of our process were highly consistent with those of the commercial software, and our process also achieved preoperative and postoperative image matching and arbitrarily selected different ROIs to obtain the real-time flow direction of each vessel. This overcomes the limitation of the software color map not being able to obtain the internal flow direction of a certain vessel.

As traditional semiquantitative blood flow analysis commercial software is embedded in the microscope, hemodynamic parameters are used to analyze intraoperative ICG-VA images to obtain the time-delayed color map that reflects the information about perfusion. Since 2011, Faber et al. (32) and Kamp et al. (33) have taken the lead in applying this commercial software to measure blood flow parameters to obtain the normal range of blood flow. Kushal et al. (34) determined the status of early arterialized veins and the relevant blood flow during arteriovenous malformation (AVM) and dural arteriovenous fistula (dAVF) surgeries. In addition, they judged whether there was sufficient relative blood flow in the branch vessels during aneurysm surgery in the case of clip-induced stenosis. Francesco et al. (14) applied this commercial software to analyze the complete intraoperative information from specific venous drainage patterns of patients and guide the decision-making process with respect to venous sacrifice, which may reduce postoperative complications. Kato et al. (35) repeatedly employed this commercial software to perform ICG-VA for each step of AVM resection with the goal of clarifying the status of feeders, drainers, and cerebral perfusion. In the above study, commercial software embedded in the microscope was used to conduct a comprehensive analysis of the entire ICG-VA image to generate static color images with data averaging. Constrained by brand and budget, this software is not widely used. In addition, the generation of high-quality images is highly dependent on video duration, environmental brightness, the coordination of ICG injection, and microscope recordings. Additionally, after ICG-VA is exported, images cannot be generated with this software independently. All these factors affect the universality and utilization of this commercial software. We proposed an automatic and quantitative blood flow analysis tool that obtains dynamic flow information. Due to the analysis of raw data, our method is not limited by the internal defects of the software or the types of microscope. Additionally, our method is an independent algorithm, and different ROIs can be selected to judge the flow direction for all vessels under the microscope, which provides more information for surgeons to make judgments.

Automatic segmentation of vessels is the first step of computer vision-aided analysis. The classical image segmentation algorithm based on machine learning uses handcrafted features and then trains a classifier to obtain the segmented images. However, the handcrafted features are singular, and the classifier performance is poor. The rapid development of deep learning provides new and effective methods for feature learning. Recently, deep learning methods have been widely used in medical image segmentation (36), among which UNet is the most commonly used for vessel segmentation (15). UNet has been extensively used in retinal vessel segmentation (37–40), 3D cerebrovascular segmentation (41–43), and cardiac vessel segmentation. Sevastopolsky et al. (44) applied UNet to segment the optic disc and cup in retinal fundus images to diagnose glaucoma. Roy et al. (45) applied UNet for retinal layer segmentation of optical coherence tomography (OCT) images. Generally, the UNet structure is perceived as a kind of encoder–decoder architecture. The encoder aims to gradually reduce the spatial dimension of feature maps and capture more high-level semantic features, while the decoder aims at restoring the details and spatial dimensions of the object. Therefore, the segmentation performance of vessels of different thicknesses can be improved by constantly capturing more high-level features with the encoder and retaining more spatial information with the decoder. To the best of the authors' knowledge, this article is the first study on the application of deep learning technology in ICG cerebrovascular image segmentation.

Image registration is the process of establishing the pixel-to-pixel correspondence between two images in the same scene. Patankar et al. (20) proposed a retinal image registration algorithm with orthogonal moment invariants as features to determine the correspondence between feature points (vessel bifurcations) in the reference and test images. Matsopoulos et al. (46) extracted the bifurcation points and then used self-organizing maps that automatically correspond to the bifurcation points in two retinal images to achieve multimodal registration. For the method based on branch registration proposed by Chen et al. (17), the branch structure consists of a main branch and three connected neighbors. However, the imperfect segmentation of vessels may affect image matching. Unlike the structure-based matching method that uses a bifurcation structure, Zana et al. (47) conducted vessel detection and then applied the Hough transform to extract features to achieve multimode registration of fundus photography. Laliberte et al. (48) combined and transformed the extracted feature points to reduce the minimum registration error. Li et al. (49) employed a rotation-invariant distance instead of the Euclidean distance to match the SIFT vectors related to key feature points. The experimental results indicated that SIFT features obtain more correct matching pairs because of their rotation invariance.

At present, due to the limited color scale, the color maps of blood perfusion obtained by commercial software cannot display the sequence of reactions with arterial fluorescent agents in the surgical field well and cannot obtain the blood flow direction in one vessel. Horn and Schunck of MIT connected a two-dimensional velocity field with the gray scale, deduced the constraint equation of optical flow, and proposed the HS optical flow method, which can calculate the motion direction of objects (22). Imbert et al. (50) applied the optical flow method to measure the direction and speed of blood flow in images of the human femoral artery. Rhode et al. (51) used the optical flow method to track the angiographic contrast agent to estimate the blood flow volume of an artery. When we used HS optical flow to measure blood flow, for the initial and final stages of the videos, it was found that the brightness changes were not significant, the values of optical flow vectors were low, and the directional characteristics were unobvious. In the middle stage of the videos, the brightness changed greatly with high direction accuracy.

However, our algorithm also has some limitations. For instance, UNet cannot be used to segment small vessels accurately. The automatic dimming mechanism of the microscope has a slight effect on the optical flow field. In the future, we will focus on reducing the missed rate of vessel segmentation, designing feature descriptors that are more robust than SIFT for registration, and determining more methods to evaluate perfusion in order to provide important clinical guidance for surgeons to perform bypass grafts more accurately.

## Data Availability Statement

The raw data supporting the conclusions of this article will be made available by the authors, without undue reservation.

## Ethics Statement

Written informed consent was obtained from the individuals for the publication of any potentially identifiable images or data included in this article.

## Author Contributions

All authors listed have made a substantial, direct and intellectual contribution to the work, and approved it for publication.

## Conflict of Interest

The authors declare that the research was conducted in the absence of any commercial or financial relationships that could be construed as a potential conflict of interest.
